# Data set for volumetric and pathological findings of epicardial adipose tissue

**DOI:** 10.1016/j.dib.2015.09.016

**Published:** 2015-09-30

**Authors:** Toshiro Kitagawa, Hideya Yamamoto, Kazuhiro Sentani, Shinya Takahashi, Hiroshi Tsushima, Atsuhiro Senoo, Wataru Yasui, Taijiro Sueda, Yasuki Kihara

**Affiliations:** a Department of Cardiovascular Medicine, Hiroshima University Graduate School of Biomedical and Health Sciences, Hiroshima, Japan; b Department of Molecular Pathology, Hiroshima University Institute of Biomedical and Health Sciences, Hiroshima, Japan; c Department of Cardiovascular Surgery, Hiroshima University Hospital, Hiroshima, Japan

## Abstract

This article contains the data regarding clinically-assessed visceral adipose tissue (VAT) area and epicardial adipose tissue (EAT) volume on computed tomography (CT) images and EAT pathology, represented by inflammation and neoangiogenesis, complementing the data reported by Kitagawa et al. [1]. In 45 patients scheduled for cardiac surgery, we studied CT images obtained prior to surgery and the numbers of CD68+ individual macrophages and CD31+ neovessels in EAT samples subsequently obtained during surgery. The data revealed a moderate correlation between VAT area and EAT volume, and a strong correlation between EAT macrophage infiltration and neoangiogenesis.

**Specifications table**

**Table:** 

Subject area	Biology
More specific subject area	Pathogenicity of human adipose tissue
Type of data	Figure, and histological image
How data was acquired	Computed tomography scan (Aquilion One; Toshiba Medical Systems, Tokyo, Japan), immunohistochemical staining, and microscope observation
Data format	Analyzed and raw data
Experimental factors	Specimens were obtained during surgery and fixed for immunohistochemistry
Experimental features	Clinical scan was performed using 320-slice computed tomography scanner, and specimens were assessed by immunohistochemical staining and analysis
Data source location	Hiroshima, Japan
Data accessibility	Data are with this article

**Value of the data**

• The data contains clinical information regarding correlations among body mass index and adipose tissue amounts.
• The data show that both inflammation and neoangiogenesis are more extensive in epicardial adipose tissue (EAT) than in paired subcutaneous adipose tissue, implying the characteristics of EAT as ‘diseased’ adipose tissue.
• The data indicate that the two biological features, inflammation and neoangiogenesis, coexist and work synergistically in increasing the EAT pathogenicity.

## 1 Data, experimental design, materials and methods

### 1.1 Study participants

We enrolled 45 patients referred for cardiac computed tomography (CT) for the investigation and diagnosis of coronary artery disease (CAD) as preparation for elective coronary artery bypass graft surgery (CABG, *n*=21) or cardiac valve surgery (non-CABG, *n*=24). In each patient, specimens of epicardial adipose tissue (EAT) adjacent to the left anterior descending (left EAT) and right coronary arteries (right EAT) were obtained during cardiac surgery (two EAT samples per patient). Additionally, subcutaneous adipose tissue (one sample per patient) was taken from the subcutaneous fat on the sternum in each patient, as control adipose tissue. These samples were subjects to later immunohistochemical analysis.

### 1.2 Clinical adipose tissue quantification

In each patient, CT scan was performed using a 320-slice CT scanner (Aquilion One; Toshiba Medical Systems, Tokyo, Japan) within 1 month prior to cardiac surgery, and visceral adipose tissue (VAT) area and EAT volume were quantified clinically. We defined VAT area as the intraperitoneal adipose tissue area with a CT density ranging from −150 to −50 Hounsfield units (HU) on plain CT images, determined from an image at the level of the umbilicus using dedicated software (Virtual Place, AZE INC., Tokyo, Japan) [2,3]. EAT volume was measured by calculating the total sum of EAT areas from 1 cm above the left main coronary artery to the left ventricular apex on images taken at 1 cm intervals: EAT was defined as adipose tissue surrounding the myocardium and limited by the epicardium on plain CT images. The EAT area of the epicardium was manually traced and defined as the area with a density range between −250 and −30 HU, which was automatically quantified using the same software as for VAT area [4].

**Fig. 1:**
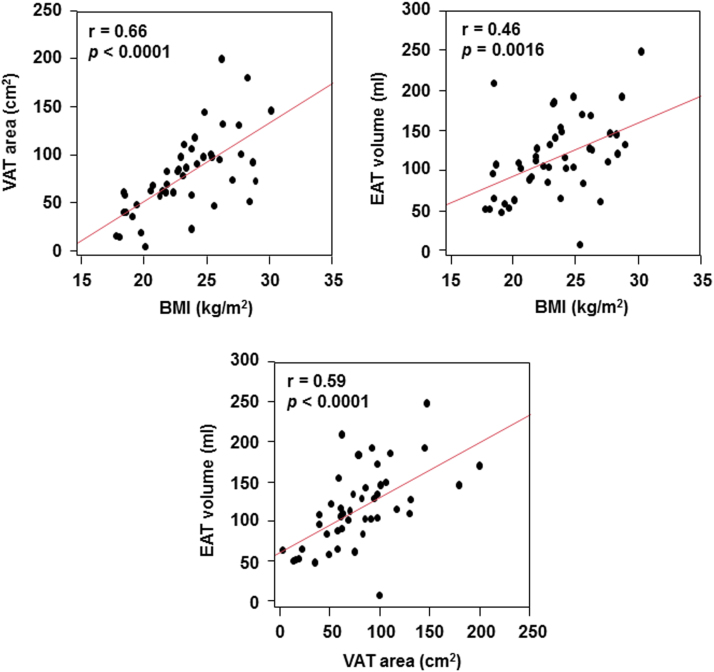
Correlations among body mass index BMI, VAT area, and EAT volume.

As a whole, VAT area and EAT volume were 79±42 cm^2^ and 116±49 mL, respectively. Fig. 1 shows correlations among body mass index (BMI) and adipose tissue amounts. Both VAT area and EAT volume had moderate correlations with BMI (*r*=0.66, *p*<0.0001, and *r*=0.46, *p*=0.0016, respectively). There was a moderate correlation between VAT area and EAT volume (*r*=0.59, *p*<0.0001).

### 1.3 Inflammation and neoangiogenesis in EAT

The adipose tissue samples (left EAT, right EAT and subcutaneous adipose tissue in each patient) were fixed in 10% buffered formalin and embedded in paraffin. A Dako Envision Kit (Dako, Carpinteria, CA, USA) was used for all immunohistochemical analyses. Antigen retrieval was performed in a citrate buffer (pH 6.0) by heating in a microwave oven (500 W) for 15 min. After endogenous peroxidase activity had been blocked with 3% H_2_O_2_–methanol for 10 min, the sections were incubated with normal goat serum (Dako, Carpinteria) for 20 min to block non-specific antibody binding sites. Immunohistochemical staining of 5-μm sections was performed with primary antibodies against CD68 (1:100, clone KP-1; Dako, Glostrup, Denmark) and CD31 (1:100, clone JC70A; Dako, Glostrup) for 60 min at room temperature, followed by incubation with peroxidase-labeled anti-mouse IgG for 60 min. Staining was completed with a 10-min incubation with the substrate–chromogen solution. Sections were counterstained with 0.1% hematoxylin. Appropriate positive and negative control samples were used.

Histology images were analyzed at a magnification of 400×. To quantify the infiltration of macrophages into the adipose tissue, CD68-positive stained individual cells were counted in three random high-power fields (1 field corresponded to a circle of radius 250 μm) in each left and right EAT specimen, and the summed number of the cells was reported for each patient. For neoangiogenesis, CD31-positive stained neovessels, rather than individual cells, in three random high-power fields were counted in each left and right EAT specimen, and the summed number of the neovessels was reported for each patient. For the quantification of subcutaneous adipose tissue, CD68-positive stained individual cells and CD31-positive stained neovessels were counted in six random high-power fields in each specimen, and were reported for each patient.

**Fig. 2:**
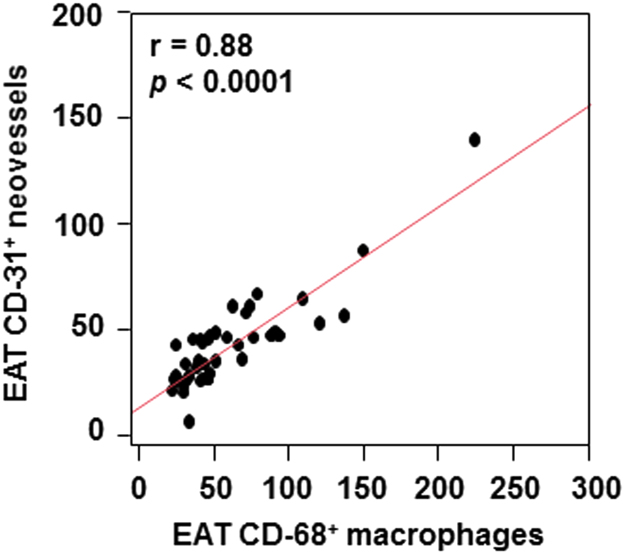
Correlation between EAT macrophage infiltration and neoangiogenesis.

**Fig. 3:**
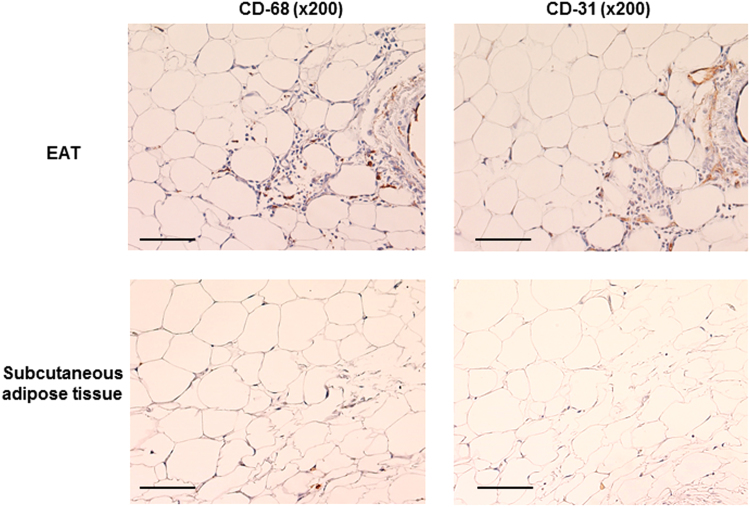
Histological examples of paired EAT and subcutaneous adipose tissues.

Macrophage infiltration was much more extensive in EAT than paired subcutaneous adipose tissue (59±40 versus 19±11, *p*<0.0001). Similarly, neoangiogenesis was much more extensive in EAT than paired subcutaneous adipose tissue (42±22 versus 15±9, *p*<0.0001). Notably, there was a strong correlation between EAT macrophage infiltration and neoangiogenesis (*r*=0.88, *p*<0.0001; Fig. 2). Fig. 3 shows example histologic images of macrophage infiltration and neoangiogenesis in paired EAT and subcutaneous adipose tissue samples.
